# Prexasertib, a cell cycle checkpoint kinases 1 and 2 inhibitor, increases *in vitro* toxicity of PARP inhibition by preventing Rad51 foci formation in *BRCA* wild type high-grade serous ovarian cancer

**DOI:** 10.18632/oncotarget.22195

**Published:** 2017-10-31

**Authors:** Ethan Brill, Takuhei Yokoyama, Jayakumar Nair, Minshu Yu, Yeong-Ran Ahn, Jung-Min Lee

**Affiliations:** ^1^ Women’s Malignancies Branch, Center for Cancer Research, National Cancer Institute, Bethesda, MD 20892, USA

**Keywords:** cell cycle checkpoint kinase inhibitor, prexasertib, LY2606368, PARP inhibitor, olaparib

## Abstract

PARP inhibitors (PARPi) have been effective in high-grade serous ovarian cancer (HGSOC), although clinical activity is limited against *BRCA* wild type HGSOC. The nearly universal loss of normal p53 regulation in HGSOCs causes dysfunction in the G1/S checkpoint, making tumor cells reliant on Chk1-mediated G2/M cell cycle arrest for DNA repair. Therefore, Chk1 is a reasonable target for a combination strategy with PARPi in treating *BRCA* wild type HGSOC. Here we investigated the combination of prexasertib mesylate monohydrate (LY2606368), a Chk1 and Chk2 inhibitor, and a PARP inhibitor, olaparib, in HGSOC cell lines (OVCAR3, OV90, PEO1 and PEO4) using clinically attainable concentrations. Our findings showed combination treatment synergistically decreased cell viability in all cell lines and induced greater DNA damage and apoptosis than the control and/or monotherapies (p<0.05). Treatment with olaparib in *BRCA* wild type HGSOC cells caused formation of Rad51 foci, whereas the combination treatment with prexasertib inhibited transnuclear localization of Rad51, a key protein in homologous recombination repair. Overall, our data provide evidence that prexasertib and olaparib combination resulted in synergistic cytotoxic effects against *BRCA* wild type HGSOC cells through reduced Rad51 foci formation and greater induction of apoptosis. This may be a novel therapeutic strategy for HGSOC.

## INTRODUCTION

High-grade serous ovarian cancer (HGSOC) is the most lethal gynecologic malignancy in the United States [1]. More than 70% of women present at an advanced stage, and recurrence is nearly universal, leading to incurable disease where treatment options remain limited [2]. Approximately 15% of women with HGSOC carry deleterious germline mutations in *BRCA1* and *BRCA2*, gene products of which are essential in homologous recombination (HR) repair for DNA double-stranded breaks (DSBs) [3]. This leaves cells dependent on other DNA damage response (DDR) proteins and pathways such as poly(ADP-ribose)polymerase 1 and 2 (PARP1 and 2), essential for the repair of DNA single-stranded breaks [4]. PARP inhibition leads to the failure of DSB repair in *BRCA1* and *BRCA2* defective cells, promoting genomic instability, apoptosis and cell death [5]. PARP inhibitor (PARPi) treatment is shown to be clinically effective in advanced HGSOC, with licensing of three FDA-approved agents to date [6-8]. Olaparib is the first licensed agent for use in heavily pretreated germline *BRCA* mutation-associated ovarian cancer [9, 10]. Only modest clinical activity has been seen with PARPi monotherapy in *BRCA* wild type HGSOC [11]. Therefore, a critical need remains for new therapeutic combination strategies that utilize the unique biology of HGSOC to increase sensitivity to PARPi.

A number of preclinical studies have attempted to sensitize HR-proficient cancer cells to PARPi by inhibiting elements in the HR DDR pathways, resulting in DNA DSBs and mitigated DNA repair [12, 13]. One such approach to modulate DNA repair activity in HGSOC is to interfere with cell cycle checkpoint signaling. An arrest of cell cycle progression is required to allow repair in the event of DNA damage and to address stalled replication forks; collapse into DSBs occurs in the absence of stabilization of stalled replication forks [14]. Essential members of cell cycle checkpoint signaling are the checkpoint kinases Chk1 and Chk2. They are activated by ATR in response to DNA replication stress or DNA damage, after which Chk1 phosphorylates and inhibits its substrates, the phosphatases CDC25C (S216) and CDC25A (S123), leading to arrest at the G2/M checkpoint [15-17].

Chk1 also plays a critical role in HR DNA repair by facilitating the BRCA2-Rad51 interaction through phosphorylation of the BRCA2 C-terminal domain and Rad51 at T309, an important step that allows transnuclear localization of the HR repair proteins in response to DSBs [18, 19]. Over-expression of Rad51 can provide resistance to DNA-damaging agents [20], which may partly explain the limited monotherapy activity of PARPi against *BRCA* wild type HGSOC. Dedes *et al.* showed a correlation between reduced Rad51 nuclear focus formation and PARPi sensitivity in PTEN-deficient endometrial cancer cell lines *in vitro* [21]. Furthermore, 96% of HGSOCs harbor a mutation in TP53 [22], thus losing control in the earlier G1/S checkpoint and making them heavily rely on Chk1-mediated G2/M cell cycle arrest for DNA repair [23]. Therefore, Chk1 is a reasonable target for a combination strategy with olaparib to maximize DDR inhibition and drive tumor cell death in treating *BRCA* wild type HGSOC.

Prexasertib mesylate monohydrate (hereafter referred to as prexasertib; LY2606368) is a selective ATP competitive small molecule inhibitor of Chk1 and Chk2 [24]. It blocks the autophosphorylation and subsequent activation of the Chk proteins, which regulate the activity of Rad51 and the CDC25 and cyclin-dependent kinases [25]. Single agent prexasertib treatment induces DNA damage and apoptosis in preclinical studies, and potential anticancer activity was shown in phase 1 clinical trials in solid tumors [26]. Prexasertib is currently being studied in phase 1/2 clinical trials as both a single agent and in combination with targeted agents or chemotherapy in adult patients with solid tumors [27]. We hypothesized that inhibiting Chk1 would sensitize *BRCA* wild type HGSOC to PARPi by preventing the formation of Rad51 foci. In this study, we aimed to evaluate the preclinical efficacy of prexasertib in combination with the PARPi olaparib in HGSOC cells at clinically attainable concentrations.

## RESULTS

### Prexasertib synergizes with olaparib to decrease cell viability in HGSOC cells

**Figure 1:**
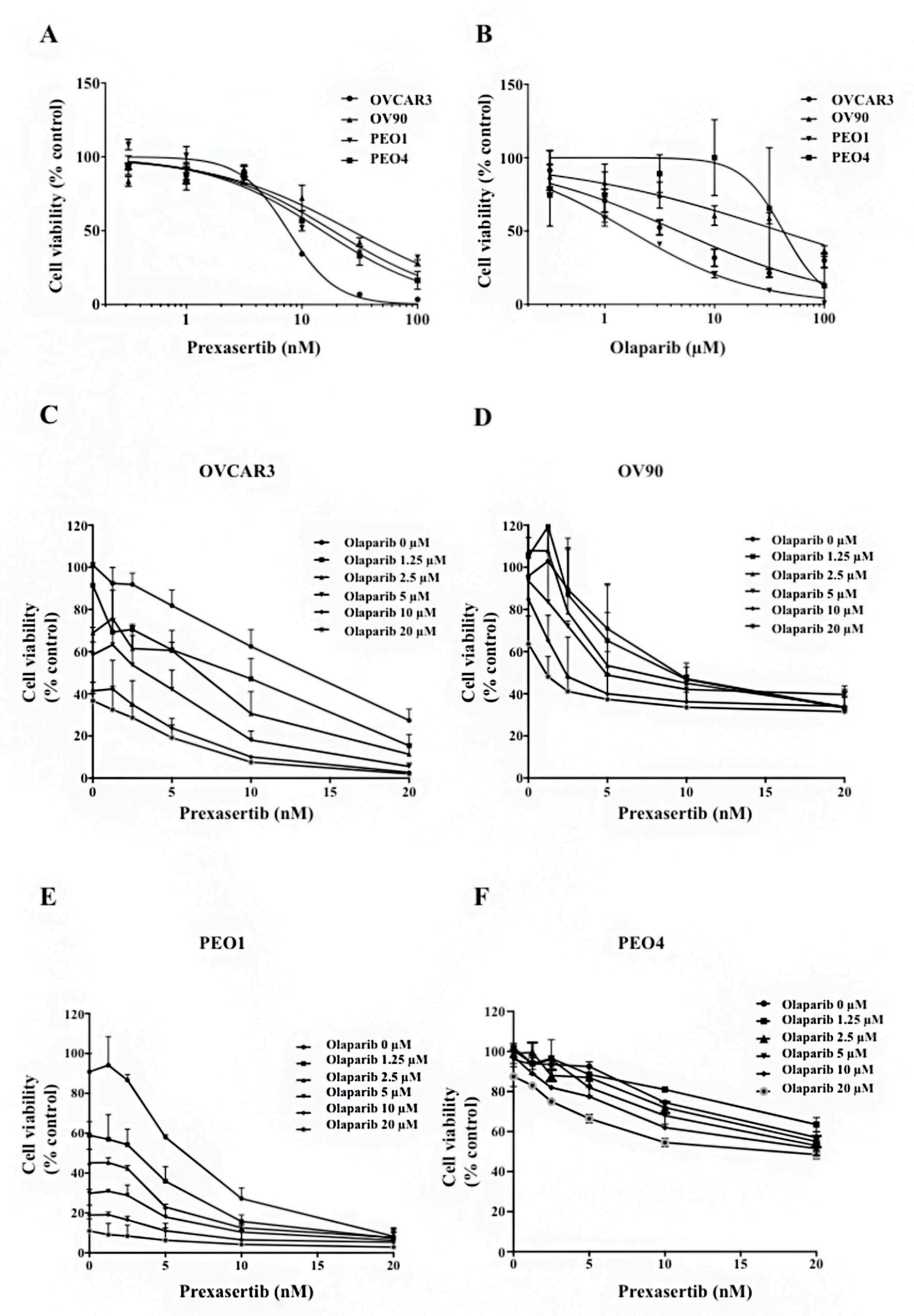
Chk1 and PARP inhibition reduces cell viability in HGSOC Cytotoxicity of prexasertib **(A)** and olaparib **(B)** was determined by XTT assay in *BRCA* mutated and *BRCA* wild type HGSOC cell lines. Cells were treated with either prexasertib (0-100 nM) or olaparib (0-100 μM) 24 hours after cells were seeded. XTT assay was performed 3 days after treatment. The cell viability was calculated relative to the 0.01% DMSO-treated control cells. The representative cell viability plots from 2 independent experiments were shown. Cells were then treated with combinations of prexasertib (0-20 nM) and olaparib (0-20 μM) 24 hours after seeding. XTT assay was performed 3 days after the combination treatment for OVCAR3 **(C)**, OV90 **(D)**, PEO1 **(E)**, and PEO4 **(F)**. The cell viability was calculated relative to the control, and was used to calculate effective combination ratios of olaparib to prexasertib and CI values as seen in Supplementary Table 1. The error bar represents the standard deviation (SD) of 3 replicates.

The cytotoxicity of prexasertib and olaparib was assessed in a panel of HGSOC cell lines. Both prexasertib and olaparib monotherapy decreased cell viability in a dose-dependent manner in both *BRCA* wild type and *BRCA* mutated cell lines (Figure 1A and 1B). PEO1 (*BRCA2* mutated) and PEO4 (*BRCA2* mutated with a gain-of-function reversion mutation) were sensitive to prexasertib, and PEO4 did not show significant loss of viability at the maximum concentration of olaparib (200 μM) used (Supplementary Figure 4: Table 1). Olaparib at clinically achievable concentrations (36- 99 μM) [28] yielded more than 50% cytotoxicity in PEO1 and OVCAR3. IC_50_ values of prexasertib in all four cell lines ranged from 6 nM to 49 nM, which were lower than clinically attainable concentrations of the recommended phase 2 dose (98-174 nM; Table 1) [26].

**Table 1:** IC_50_ values of prexasertib and olaparib in HGSOC cells. Cell viabilities were calculated relative to the control, and IC_50_ values were determined by Prism and the mean ± SD of 2 independent experiments were shown.

Cell Line	Olaparib (μM), mean ± SD	Prexasertib (nM), mean ± SD
**OVCAR3**	6.18 ± 2.16	6.34 ± 1.8
**OV90**	57.00 ± 24.65	35.22 ± 11.71
**PEO1**	2.04 ± 0.49	12.65 ± 8.27
**PEO4**	ND	48.79 ± 3.89

ND: Not determined. PEO4 did not show significant loss of viability (81-89% viable cells) at the maximum concentration of olaparib (200 μM) used in XTT assay.

We next assessed synergy of the combination treatment and the conditions under which such synergism occurred. Cell viability curves for combination treatments ranging from 0-20 μM for olaparib and 0-20 nM for prexasertib were determined from XTT assays (Figure 1C-1F). We selected two dose combinations of prexasertib/olaparib (5nM/5μM and 10nM/10μM) to test for synergism. Synergism was assessed by using Combination Index (CI) values as determined by Chou and Talalay (Supplementary Table 1) [29]. A combination of 5nM/5μM gave CI values that were <1 for all cell lines tested, and a combination of 10nM/10μM gave CI values that were <1 for all cell lines tested except OV90 (Supplementary Table 1). A prexasertib/olaparib combination dose of 20nM/20μM gave CI values that were <0.3 for OVCAR3, OV90 and PEO1, suggesting strong synergism, and were extremely cytotoxic (cell viability <40%). Because lower cytotoxicity would ensure that enough remaining viable cells would be available for subsequent assays, a combination of 5nM/5μM prexasertib/olaparib was chosen to further study the underlying molecular mechanisms that may drive synergism.

### Prexasertib does not affect PAR incorporation and olaparib does not alter Chk1 activity

**Figure 2:**
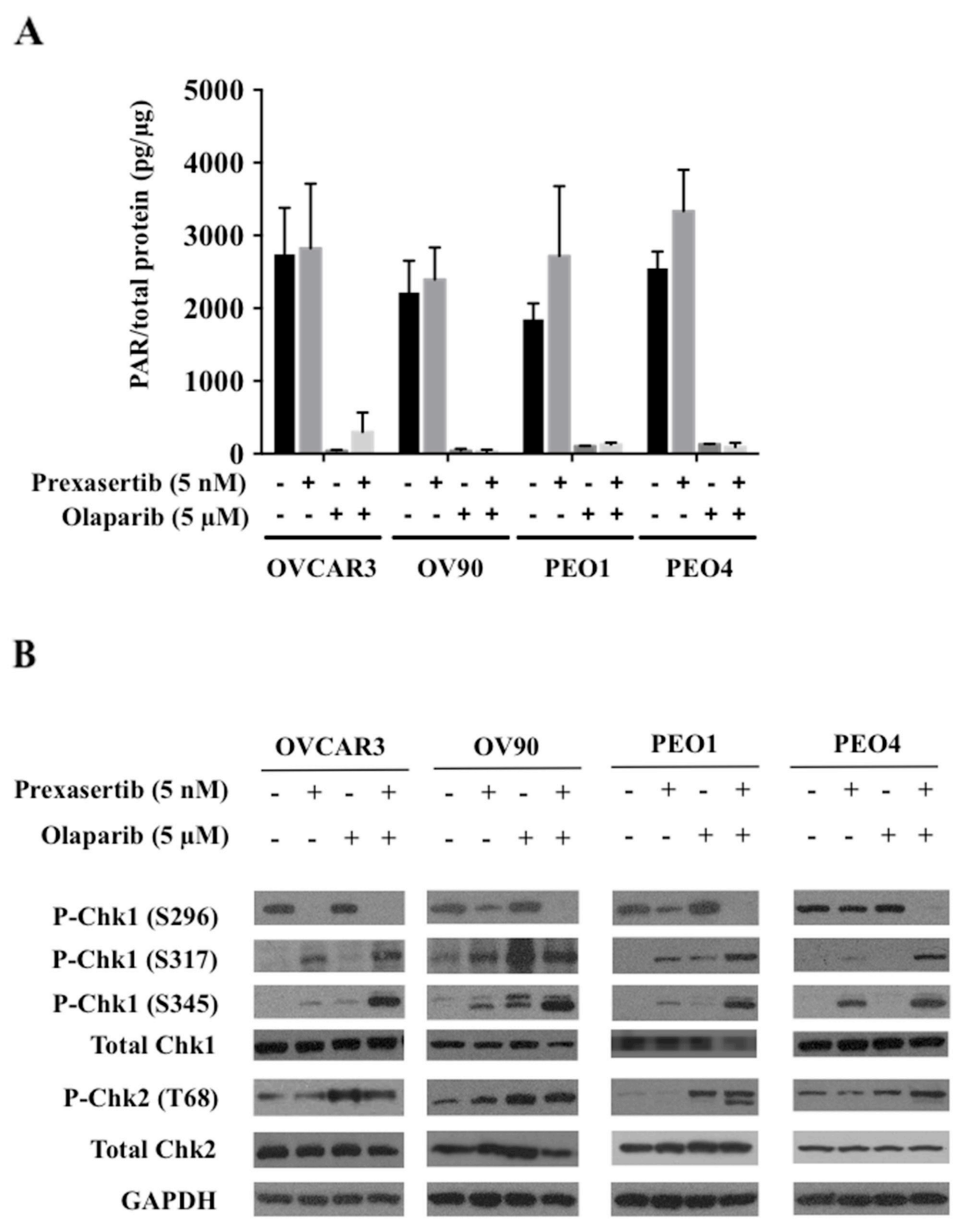
Prexasertib and olaparib display on-target effects at lower than clinically achievable doses **(A)** Olaparib’s effect on PARP1 activity was measured by assessing decreases in PAR levels. The experiment was repeated twice, with each experiment having two replicates. The mean ± SD of 2 independent experiments was shown. **(B)** Prexasertib’s inhibitory effect on Chk1 and Chk2 was assessed by immunoblotting. The representative immunoblot images were shown. GAPDH was used as a loading control.

We first examined on-target effects of each monotherapy treatment and their combination. Olaparib significantly reduced PAR incorporation in all cell lines both in monotherapy and in combination, and prexasertib neither stimulated nor inhibited PAR incorporation (Figure 2A). Chk1 and 2 protein phosphorylation was examined to investigate prexasertib target effects. Phosphorylation of S317 and S345 Chk1 reflect ATR activation in response to DNA damage, and S296 Chk1 is the activating autophosphorylation site, essential for downstream Chk1 phosphorylation activity [17, 30, 31]. Prexasertib monotherapy decreased S296 autophosphorylation and increased Chk1 phosphorylation at S317 and S345 compared to the control in all cell lines except PEO4. Olaparib monotherapy did not significantly reduce S296 phosphorylated Chk1 levels. Combination treatment inhibited S296 Chk1 autophosphorylation while it triggered greater Chk1 S345 and S317 phosphorylation than either monotherapy in all cell lines tested (Figure 2B). Treatment with olaparib as a monotherapy and in combination with prexasertib increased phosphorylation of Chk2 (T68) in all cell lines. Total Chk1 and Chk2 expression did not change upon either monotherapy treatment for all cell lines, but did decrease in the combination treatment for OV90 (Figure 2B). The Chk1 downstream proteins CDC25A and CDC25C showed no changes (Supplementary Figure 3B).

### Chk1 inhibition prevents nuclear Rad51 foci formation in response to olaparib treatment

**Figure 3:**
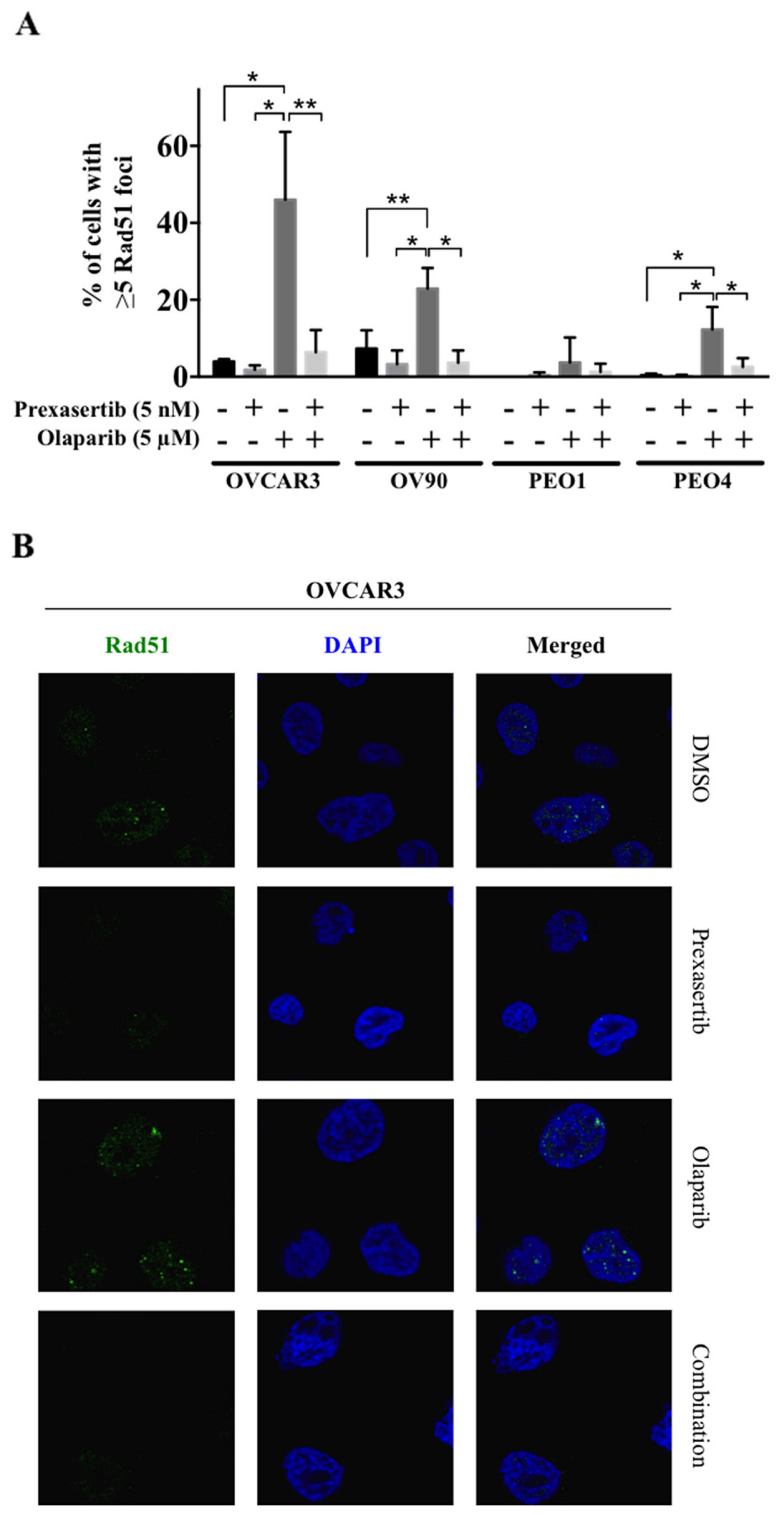
Chk1 inhibition suppresses the nuclear Rad51 foci formation in response to olaparib treatment **(A)** Percent of cells with more than 5 Rad51 foci was determined. The data was presented as the mean ± SD of 3 independent experiments. The statistical significance was analyzed using one-way ANOVA. (^*^ = p < 0.05, ^**^ = p < 0.01). **(B)** Confocal microscopic images of OVCAR3 cells were shown as representative images.

To examine our hypothesis of synergistic cytotoxicity by reduced Rad51 response, the extent of Rad51 focus formation was assessed by immunofluorescence. Olaparib treatment induced nuclear Rad51 foci formation in *BRCA* wild type HGSOC cell lines and PEO4, a *BRCA*2 gain-of-function revertant cell line (Figure 3), while prexasertib had no impact on nuclear Rad51 foci formation. The induction of nuclear Rad51 foci by olaparib was almost completely abrogated when Chk1 was inhibited by prexasertib in all *BRCA* wild type HGSOC cell lines and PEO4 (Figure 3 and Supplementary Figure 2).

### Prexasertib and olaparib induce greater DNA damage in combination

**Figure 4:**
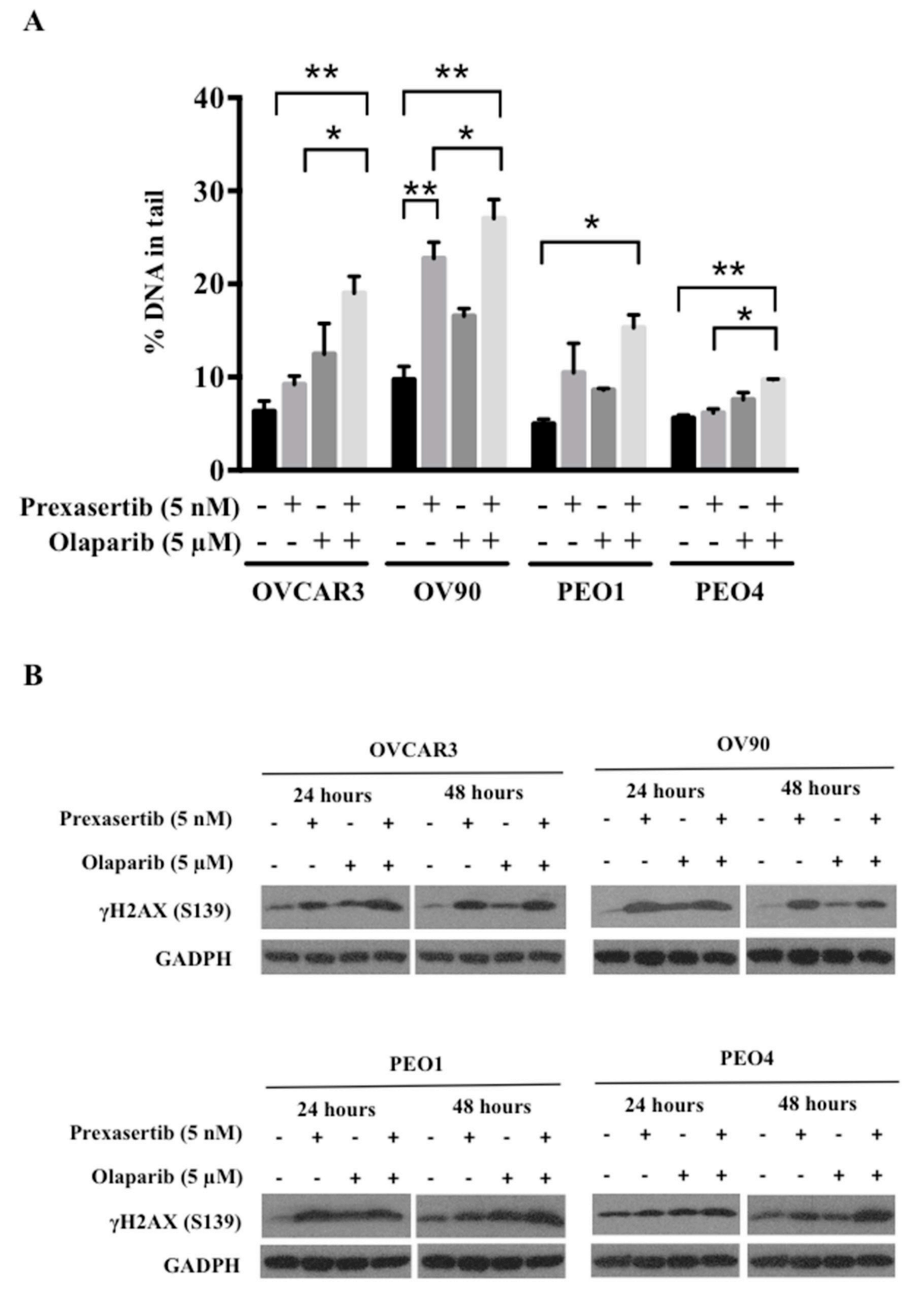
Prexasertib and olaparib cause DNA damage in HGSOC cells DNA damage was assessed with alkaline comet assay **(A)** and immunoblotting **(B)**. (A) The percentage of DNA in comet tails significantly increased by the combination treatment compared to the control in all cell lines (p<0.05). The experiment was repeated 3 times and the data was presented as the mean ± SEM. The statistical significance was analyzed using one-way ANOVA (^*^ = p < 0.05, ^**^ = p < 0.01). (B) γH2AX (S139) immunoblotting was performed with total lysates for 24 and 48 hours after treatment. The representative immunoblot images were shown. GAPDH was used as a loading control.

Inhibition of either Chk1 or PARP is known to cause DNA damage and dysregulation of DNA repair [32, 33]. We next examined if the observed greater cytotoxicity in the combination therapy was induced due to increased DNA damage. Significantly increased DNA damage occurred with the combination, demonstrated by greater comet tail DNA percentage, compared to the control conditions (p<0.05, Figure 4A). Damage incurred by either single agent alone was not significantly different from the control in each cell line (Figure 4A) except for OV90, whereas significantly increased DNA damage was observed in the prexasertib treatment compared to the control (p<0.01, Figure 4A). Increased γH2AX (S139) expression was also seen with the combination treatment at 24 and 48 hours (Figure 4B). We performed γH2AX (S139) immunofluorescence confocal imaging to further elucidate DNA damage induced by the combination treatment. We found a significant increase in γH2AX focus formation compared to the control in OVCAR3 and PEO4 (p<0.05; Supplementary Figure 1). Pan-nuclear γH2AX staining was observed in prexasertib and in combination treated cells, suggesting both prexasertib alone and in combination induced greater DNA damage and suspected apoptosis than olaparib alone [34].

### Combination treatment yields greater apoptosis than either monotherapy in HGSOC cells

**Figure 5:**
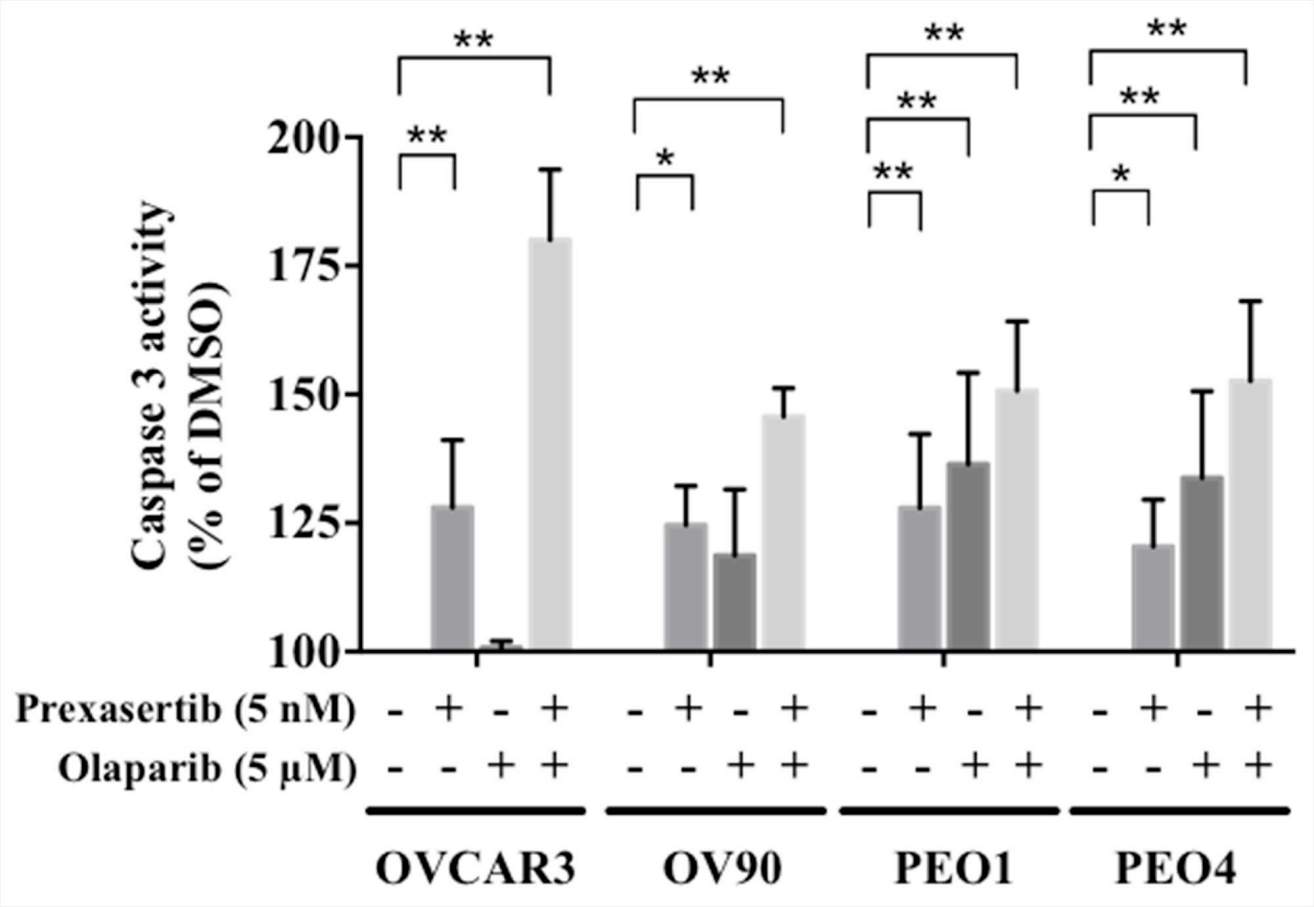
Combination treatment increases apoptosis in HGSOC The caspase 3 activity in each condition was calculated relative to the control. The experiment was performed in duplicate and repeated twice. The data is presented as the mean ± SD of 2 independent experiments. The statistical significance was analyzed using multiple comparison t tests (^*^ = p < 0.05, ^**^ = p < 0.01).

We next examined whether the decreased cell viability was attributable to greater apoptosis. We observed greater caspase 3 activity with the combination treatment compared to the control in all cell lines (Figure 5), suggesting the combination treatment induced greater apoptosis and cell death in both *BRCA* wild type and *BRCA* mutated HGSOC cell lines.

### Chk1 inhibition perturbs G2/M cell cycle arrest induced by olaparib

**Figure 6:**
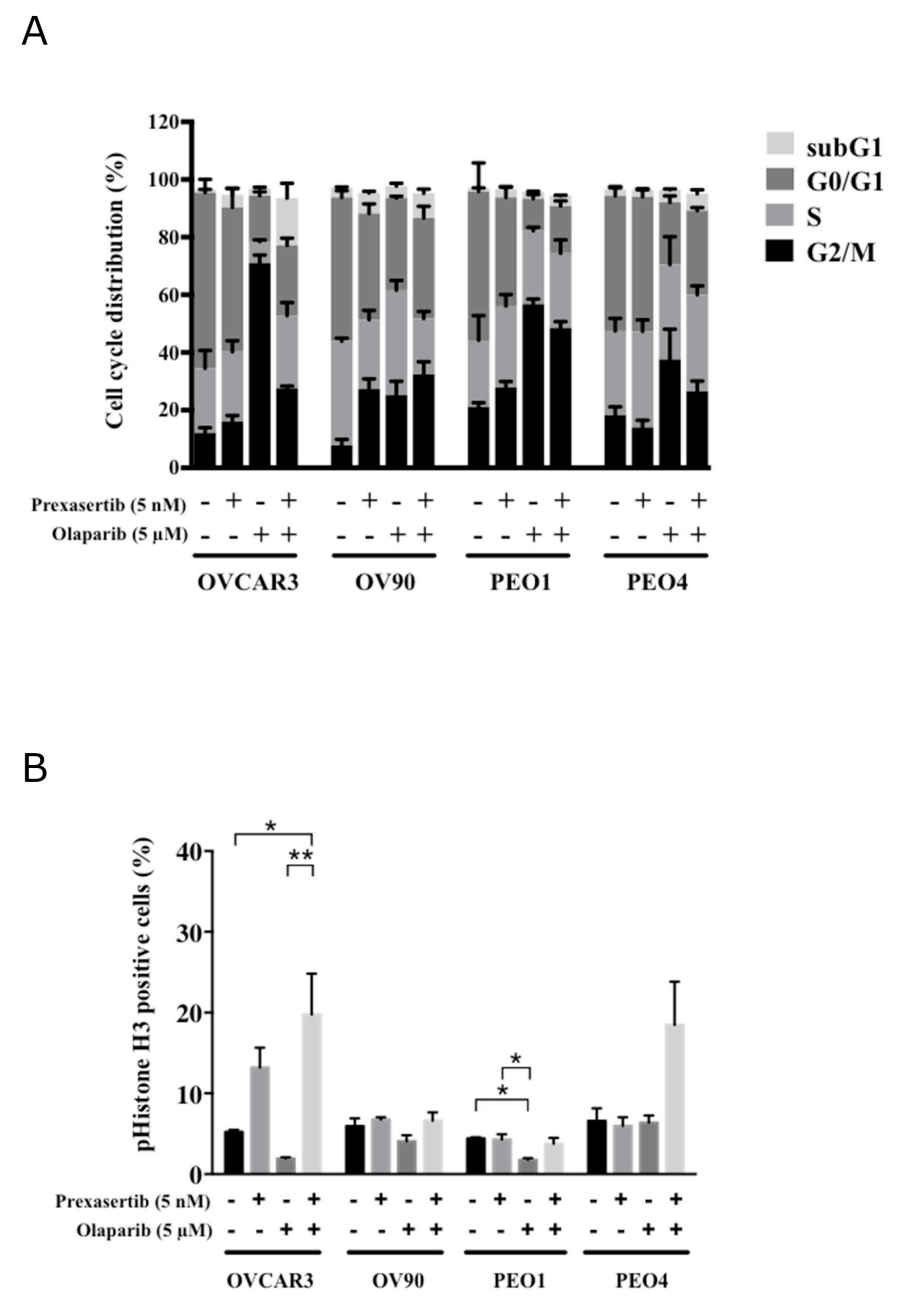
Prexasertib perturbs the cell cycle **(A)** The mean ± SD of 3 independent experiments of cell cycle analysis via flow cytometry was shown. **(B)** The percentage of phospho(p)-Histone H3 (S10) positive cells relative to the total number of cells was analyzed by flow cytometry 48 hours after treatment (^*^ = p < 0.05, ^**^ = p < 0.01).

Chk1 is essential in arresting DNA damaged cells at the G2/M checkpoint. We investigated how this perturbation could be contributing to the observed synergism. Cells treated with olaparib monotherapy showed enrichment in the G2/M phase compared to DMSO treated cells except for OV90 (Figures 6A; Supplementary Figure 3A). Prexasertib alone did not change the cell cycle distribution in all cell lines except in OV90. Cells in the G2/M phase were reduced in the combination treatment compared to olaparib monotherapy in these three cell lines: from 73.3% to 43.7% for OVCAR3 (p<0.001), 59.4% to 51.5% for PEO1 (p=0.018) and 39.0% to 27.9% for PEO4 (p=0.018) (Figure 6A). Phospho(p)-Histone H3 levels also increased in the combination treatment compared to olaparib alone in OVCAR3 (Figure 6B), indicating that prexasertib in the combination treatment forced the olaparib-induced arrested cells to enter M phase. Collectively, these results support the idea that prexasertib overrides olaparib-induced G2/M arrest.

## DISCUSSION

Inhibition of the ATR/Chk1 axis has been reported to cause replication catastrophe, DNA damage and cell death [35], making this pathway an attractive target for a combination strategy with the PARPi olaparib in HGSOC [36]. We demonstrated the synergistic cytotoxicity of prexasertib and olaparib combination against both *BRCA* wild type and *BRCA* mutant HGSOC cell lines at clinically attainable concentrations. PARP inhibition is associated with induction of Rad51 nuclear accumulation and focuses to sites of DNA damage for initiation of repair [37]. Prexasertib, with little direct effect on Rad51 focus formation by itself, abrogated olaparib-induced Rad51 focus formation, resulting in greater DNA damage that was measured by several means. The drug concentrations used in this study were carefully selected to perform a clinically relevant *in vitro* study given both drugs cause significant myelotoxicity in humans [26, 38]. In a phase 1 study by Hong *et al.*, prexasertib resulted in grade 4 neutropenia in 73% of patients with advanced solid tumors, which may become a challenge for clinical investigations when combined with cytotoxic chemotherapy or DNA repair inhibitors such as PARPi. We proceeded using doses of prexasertib and olaparib at clinically attainable concentrations, which are below the concentrations achieved by the recommended phase 2 doses for each drug (36 μM for olaparib and 98 nM for prexasertib) [26, 28].

One of the major cellular HR responses to DNA damage includes the nuclear recruitment of the BRCA2-Rad51 complex [20, 39]. Rad51 translocates into the nucleus upon DNA damage, forming foci and assisting the DNA strand-pairing step of HR [40, 41]. Rad51 suppression via microRNA-506 has been shown to sensitize serous ovarian cancer to DNA damaging drugs such as cisplatin and PARPi [42]. Low quantities of Rad51 foci measured by immunofluorescence in post-chemotherapy biopsies were also associated with pathologic complete responses to anthracycline-based chemotherapy in sporadic primary breast cancers [43]. Additionally, reduced levels of Rad51 expression by siRNA are shown to increase sensitivity to PARPi in human gastric cancer cells, and restoration of Rad51 via plasmid transfection attenuated drug sensitivity [44]. Recently, Narayanaswamy and colleagues reported prexasertib blocked nuclear localization of Rad51 in pancreatic cancer cell lines in response to DNA damage by gemcitabine [45]. A Wee-1 inhibitor, AZD1775, also attenuated Rad51 nuclear localization in pancreatic cancer cells, and resulted in sensitization to DNA damaging effects from radiation and PARPi [12]. We observed increased nuclear Rad51 foci levels by olaparib treatment in non-*BRCA* mutated HGSOC cell lines, and these levels were significantly diminished in the combination treatment. Furthermore, the combination treatment caused greater apoptosis relative to the control in all *BRCA* wild type cell lines. The PARPi olaparib has shown clinical benefit both in *BRCA* mutant and wild type HGSOC, although response is reduced in recurrent *BRCA* wild type HGSOC [9]*.* Thus, our findings suggest a potential benefit of the combination therapy in non-*BRCA* mutated HGSOC by attenuation of DNA repair activity and greater apoptosis.

Other preclinical studies have provided evidence that the combination of Chk1 inhibitors and PARPi result in increased γH2AX phosphorylation and apoptosis in mammary carcinoma cells [46]. The combination therapy that we used demonstrated increased cytotoxicity by causing DNA damage, inhibiting Rad51 transnuclear localization and abrogating critical cell cycle checkpoints in *BRCA* wild type HGSOC [42]. Kim *et al.* recently demonstrated that combination of the ATR inhibitor AZD 6738 with olaparib caused an accumulation in chromosomal breakage, abrogation of the G2/M cell cycle checkpoint and increased apoptosis in *BRCA2* mutated ovarian cancer cells [47]. Similar to our results, they also found that the combination of the Chk1 inhibitor MK8776 with olaparib was synergistically cytotoxic in PEO1 cells, but not in PEO4 cells [47]. Our results show Rad51 foci formation was inhibited by prexasertib, indicating that decreased efficiency of HR DNA repair contributed to the sensitization of the cell lines to olaparib.

Accumulated DNA damage is a key factor resulting in greater cell death and possible synergistic cytotoxicity of olaparib with Chk1 or ATR inhibitors [48]. Sen *et al.* demonstrated the synergistic cytotoxicity of prexasertib and olaparib in small cell lung cancer, documenting increased γH2AX expression and apoptotic cell death [49]. It has been reported that DNA damage and replication stress induce γH2AX phosphorylation, either with separate foci formation in damaged cells or pan-nuclear staining in apoptotic cells by immunofluorescence [50]. Consistent with this, our findings showed γH2AX foci formation in olaparib-treated cells. Pan-nucleic γH2AX staining was observed in prexasertib and in the combination treated cells, suggesting prexasertib induced greater apoptosis and replication stress than olaparib alone [51-53].

Most tumor cells largely rely on the G2/M checkpoint for DNA damage response because of a lack of G1/S checkpoint function, due to aberrant p53 function [54]. Our study confirms olaparib induces HGSOC cells to arrest in the G2/M phase as previously demonstrated [55]. This G2/M arrest was abrogated by prexasertib treatment in HGSOC cell lines. Thus, it is plausible that the combination of olaparib and prexasertib largely inhibits the function of the G2/M checkpoint, and speeds up the cell cycle, forcing cells with unrepaired DNA damage into mitosis and eventually leading to apoptosis and cell death [56], more dominantly in the *BRCA* wild type cell lines.

Prexasertib monotherapy is currently under clinical investigation and demonstrates early clinical activity in women with recurrent HGSOC (NCT02203513) [57]. Transient grade 4 neutropenia has been reported in 73% of prexasertib-treated patients, but clinically significant febrile neutropenia was rare [26, 57]. PARPi as a class also result in reduction of neutrophils [58], although this class of agents has been documented to have activity at submaximal doses when used in combination treatments [47]. Synergistic drug combinations carry the expectation of greater therapeutic efficacy, although they may also increase the severity of adverse effects. In addition, an ongoing phase 1 study of prexasertib and olaparib in advanced solid tumors (NCT03057145) and phase 2 study of prexasertib in solid tumors with replication stress or HR deficiency (NCT02873975) will reveal greater insight into the possible mechanisms driving clinical activity, as well as strategies for chemotherapeutic combinations that exploit such mechanisms.

Here we demonstrated the synergistic cytotoxicity of the combination treatment of prexasertib and olaparib against HGSOC. Prexasertib treatment increased cell cycle replication stress, and impaired Rad51 foci formation induced by olaparib in the combination treatment. Our study supports further evaluation of the therapeutic potential of the combination treatment including a Chk1 inhibitor and PARPi for *BRCA* wild type HGSOC.

## MATERIALS AND METHODS

### Cell lines

OVCAR3 (*BRCA* wild-type) cells were obtained from ATCC (Manassas, VA, USA), and OV90 (*BRCA* wild-type), PEO1 (*BRCA1* wild-type and *BRCA2* mutated 4035T>C) [47, 59], and PEO4 (*BRCA1* wild-type and *BRCA2* mutated with gain-of-function mutation) cells were gifted by Dr. Annunziata (National Cancer Institute; Bethesda, MD, USA) [60-62]. The authentication of all cell lines was performed at the Frederick National Laboratory for Cancer Research on March 18th, 2016. 15 STR markers (D8S1179, D21S11, D7S820, CSF1P0, D3S1358, TH01, D13S317, D16S539, D2S1338, D19S433, vWA, TPOX, D18S51, D5S818, and FGA) and amelogenin for gender determination were tested to determine unique identity. All cell lines were grown in RPMI 1640 medium with (+) L-glutamine supplemented with 10% fetal bovine serum and 1% Penicillin/Streptomycin.

### Drug preparations

Stock solutions of 100 mM olaparib (AZD2281) (Selleck Chemicals; Houston, TX, USA, Cat No. S1060) and 100 μM prexasertib (LY2606368) (Eli Lilly; Indianapolis, IN, USA; MTA in disclosures) were prepared in dimethylsulfoxide (DMSO) and stored in aliquots at -80 °C. Treatment solutions with concentrations of 5 nM and 5 μM for prexasertib and olaparib, respectively, were prepared by diluting stock solutions in cell culture medium.

### Cell viability (XTT) assay

Cell viability was assessed by the Cell Proliferation Kit II (XTT assay) (Roche; Indianapolis, IN, USA) according to manufacturer’s instructions. 2000 cells/well were seeded in 96-well plates and treated with either olaparib (0-100 μM), prexasertib (0-100 nM), both or 0.01% DMSO 24 hours after seeding. Cells were treated for 3 days, and the absorbances were measured by SpectraMax M5 (Molecular Devices, Sunnyvale, CA, USA). Cell viability was calculated relative to DMSO-treated control cells. Based on the dose-response curves plotted from the relative absorbance values, IC_50_ values were calculated using GraphPad Prism v. 7.0 (GraphPad Software Inc., La Jolla, CA, USA) (Table 1). Combination Index (CI) values were calculated using the Compusyn software (ComboSyn Inc., Paramus, NJ, USA). CI values less than 1 indicate synergism [63].

### PAR concentration assay

Cells were seeded at 2000 cells/well were seeded in 96-well plates and treated with either 5 μM olaparib, 5 nM prexasertib, both or 0.01% DMSO for 48 hours, and 50 μg of the lysate were used in the analysis. PAR levels in cellular lysates were measured using a PARP *in vivo* Pharmacodynamic Assay II (Trevigen) per the manufacturer’s instructions. PAR level was calculated relative to the 0.01% DMSO-treated cells.

### Immunoblotting

Cells were seeded at 2000 cells/well in 96-well plates and treated with either 5 μM olaparib, 5 nM prexasertib, both or 0.01% DMSO for 48 hours, and subjected to immunoblotting [64]. The following antibodies were used: CDC25C (Cell Signaling Technology, #4688; Danvers, MA, USA), S216-pCDC25C (Cell Signaling, #9528), T48-pCDC25C (Cell Signaling, #9527), CDC25A (Cell Signaling, #3652), S124-pCDC25A (Abcam, #ab156574), CDC2 (Cell Signaling, #9112), Y15-pCDC2 (Cell Signaling, #9111), Chk1 (Cell Signaling, #2360), S296-pChk1 (Cell Signaling, #2349), S317-pChk1 (Cell Signaling, #2344), S345-pChk1 (Cell Signaling, #2341), Chk2 (Cell Signaling, #2662), T68-pChk2 (Cell Signaling, #2197), GAPDH (Cell Signaling, #2118), S139-γH2AX (Abcam, #ab11174), ECL goat anti-mouse IgG HRP and ECL goat anti-rabbit IgG HRP (Cell signaling, #7076 and #7074).

### Immunofluorescence confocal microscopy (IF)

Cells were grown on 12mm poly L-Lysine-coated coverslips (Corning Inc., Oneonta, NY, USA) and treated with either 5 μM olaparib, 5 nM prexasertib, both or 0.01% DMSO. OVCAR3, OV90, and PEO1 were treated for 48 hours while PEO4 was treated for 72 hours. Cells were fixed in 4% paraformaldehyde for 10 minutes, permeabilized with 0.25% Triton-X, and blocked with 1% BSA in PBS. Cells were incubated in primary and secondary antibodies for 1 hour each. For primary antibodies, anti-rabbit Rad51 (Santa Cruz Biotechnology, Santa Cruz, CA, #8349) at 1:50 ratio or anti-human γH2AX (Abcam; Cambridge, MA, USA, #11174) at 1:600 ratio in 1% BSA were used. Alexa Fluor 488nm (Invitrogen, Carlsbad, CA) goat anti-rabbit secondary antibodies (1:100) and Alexa Fluor 647nm (Invitrogen) goat anti-mouse secondary antibodies (1:200) were used for Rad51 and γH2AX detection. The slides were mounted with Vectashield mounting medium with DAPI (Vector Labs, Burlingame, CA, USA), and images were collected with the LSM 780 confocal microscope with a 63x/1.4 oil immersion objective. The number of Rad51 foci was quantified in more than 100 cells per condition using the Focinator software [65]. Rad51 foci positive cells were defined as cells with more than 5 foci in the nucleus [12]. For γH2AX staining, cells with γH2AX foci formation were categorized as having 5-9, 10-14 or >15 foci per nucleus, or having pan-nuclear staining [66].

### Comet assay

Cells were seeded in 6-well plates at a range of 2-3x10^5^ cells/well, and treated with 5 μM olaparib, 5 nM prexasertib, both or 0.01 % DMSO for 48 hours. DNA double-strand breaks were measured by alkaline comet assay according to the manufacturer’s instruction (Trevigen; Gaithersburg, MD, USA). Stained cells were imaged with a Nikon Diaphot microscope. The percentage of DNA in tail was measured in more than 50 cells/condition with CometScore Pro (TriTek Corporation; Sumerduck, VA, USA). Three independent experiments were performed for each condition.

### Caspase 3 activity assay

Cells were seeded in 6-well plates at 3 x 10^5^ cells/well and treated with 5 μM olaparib, 5 nM prexasertib, both or 0.01% DMSO for 24 hours and 48 hours, and 50 μg of the lysate were used in analysis. Caspase 3 activity was examined using a CaspACE Assay System, Colorimetric (Promega; Madison, WI, USA) according to the manufacture’s instruction. Caspase 3 activity was calculated relative to the control.

### Flow cytometry

Cells were seeded in 6-well plates at 3x10^5^ cells/well and treated with 5 μM olaparib, 5 nM prexasertib, both or 0.01% DMSO. Cells were treated with indicated drugs for 24 hours and 48 hours for cell cycle analysis. Briefly, cells were fixed in 4% paraformaldehyde for 15 minutes, permeabilized with 0.25% Triton-X for 5 minutes, and blocked with 10% goat serum in PBS for 30 minutes. Cells were incubated in primary and secondary antibodies for 1 hour each at 4°C. Cellular DNA was stained using 7-AAD. For primary antibodies, anti-mouse phospho-Histone H3 (Ser10) (Cell Signaling, #9701) at 1:50 ratio in 10% goat serum/PBS was used. Alexa Fluor 488nm goat anti-rabbit secondary antibodies (Invitrogen) and Alexa Fluor 647nm goat anti-mouse secondary antibodies (Invitrogen) were used at 1:500 dilutions. Cell cycle analysis was performed using the BD Pharmingen APC BrdU flow kit according to the manufacturer’s protocol (BD Biosciences; San Jose, CA, USA). Stained cells were collected with a FACScalibur (BD Biosciences) and analyzed using the FlowJo v. X.0.8 software (Treestar; Ashland, OR, USA).

### Statistical analysis

The data were subjected to one-way ANOVA with Tukey post-comparison tests and multiple comparison t tests in GraphPad Prism v. 7.0 (GraphPad Software). All differences were considered statistically significant if p<0.05.

## SUPPLEMENTARY MATERIALS FIGURES AND TABLE




