# Supplementary material for: Prexasertib, a cell cycle checkpoint kinases 1 and 2 inhibitor, increases in vitro toxicity of PARP inhibition by preventing Rad51 foci formation in BRCA wild type high-grade serous ovarian cancer
Source: Oncotarget. 2017 Oct 31;8(67):111026–40. doi: 10.18632/oncotarget.22195 (PMC5762302; doi:10.18632/oncotarget.22195)
Supplement: Supplementary file 1 [file oncotarget-08-111026-s001.pdf]

Prexasertib, a cell cycle checkpoint kinases 1 and 2 inhibitor, increases *in vitro* toxicity of PARP inhibition by preventing Rad51 foci formation in *BRCA* wild type high-grade serous ovarian cancer

SUPPLEMENTARY MATERIALS

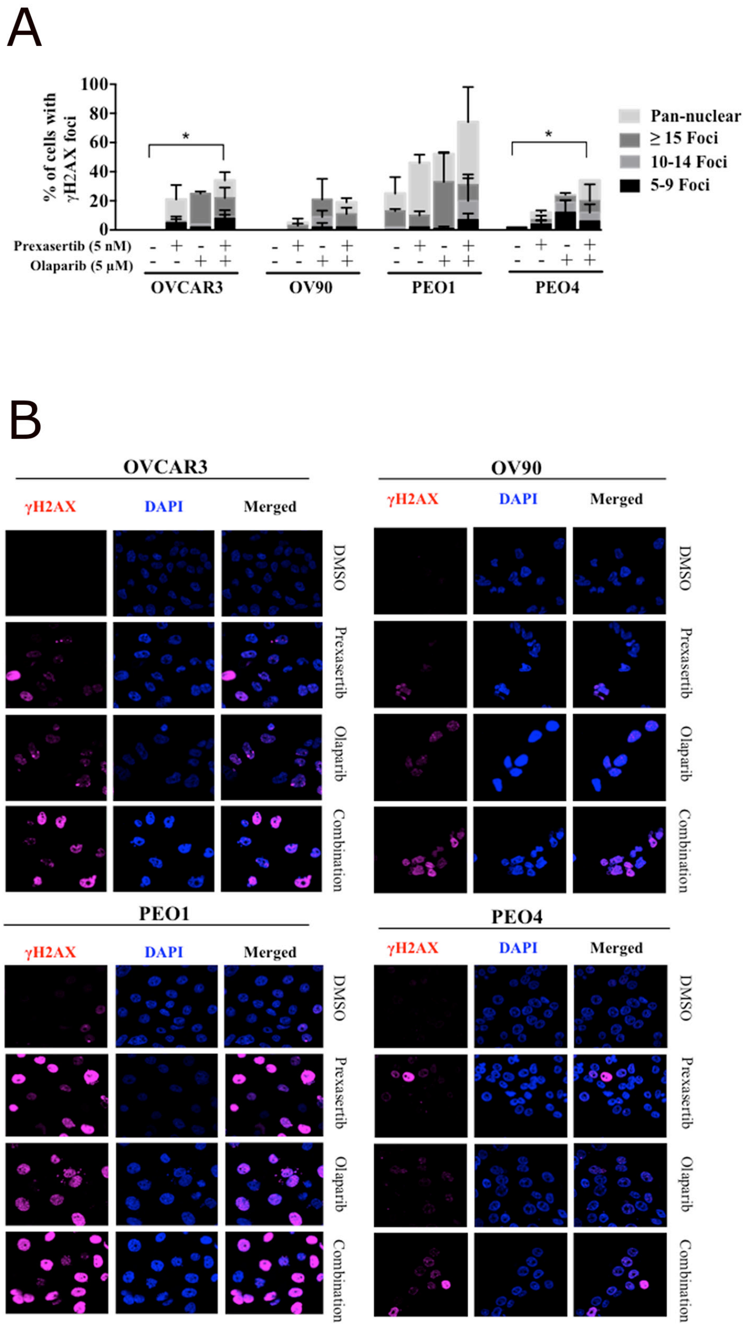

Supplementary Figure 1: Immunofluorescent confocal microscopic analysis of γH2AX. (A) The number of γH2AX foci per nuclei was determined from the images, with the percentage of foci formation from 50 cells. The statistical significance was analyzed using one-way ANOVA. (* = $p < 0.05$). **(B)** All images were taken at x 63 magnification. The representative images from 3 independent experiments were shown. Cell nuclei were stained with DAPI.

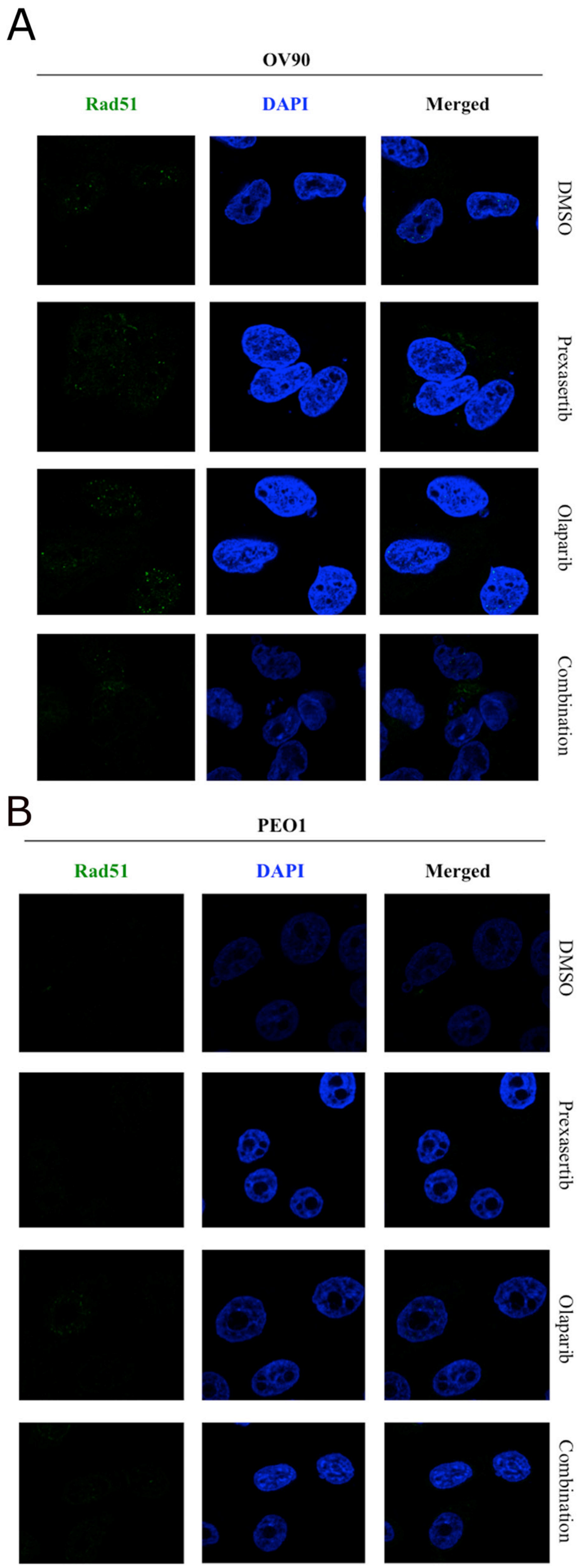

Supplementary Figure 2: Chk1 inhibition suppresses the nuclear Rad51 foci formation in all BRCA wild type HGSOC cell lines. (A-C) OV90, PEO1 and PEO4 cells were treated with 5 nM prexasertib, 5 μM olaparib, both or 0.01% DMSO for 48 hours, and PEO4 cells were treated for 72 hours. Cells were subjected to immunofluorescent staining with Rad51 antibodies. Images were taken at x 63 magnification. ***(Continued)***

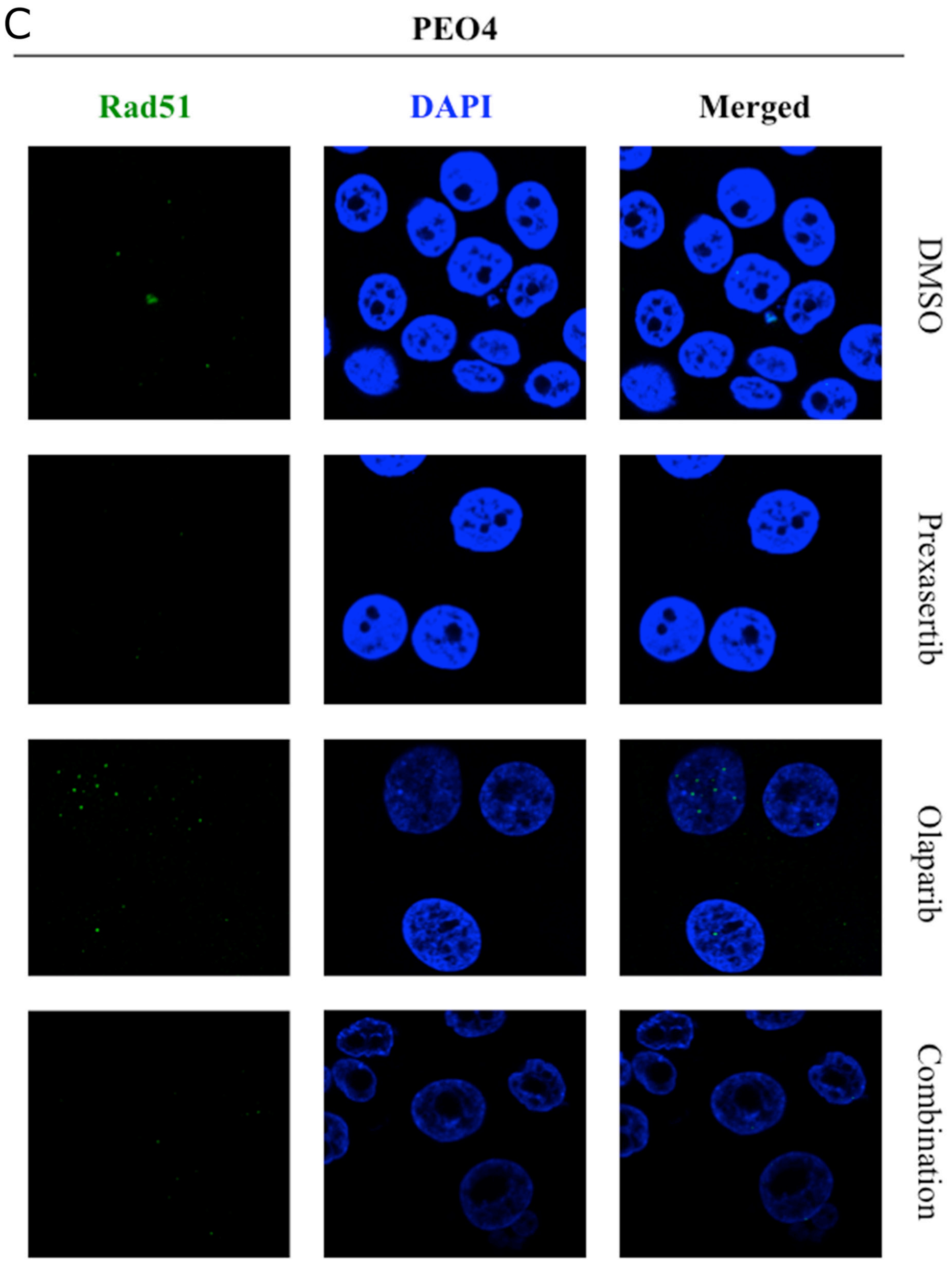

Supplementary Figure 2 ***(Continued)*****: Chk1 inhibition suppresses the nuclear Rad51 foci formation in all BRCA wild type HGSOC cell lines. (A-C)** OV90, PEO1 and PEO4 cells were treated with 5 nM prexasertib, 5 μM olaparib, both or 0. 01% DMSO for 48 hours, and PEO4 cells were treated for 72 hours. Cells were subjected to immunofluorescent staining with Rad51 antibodies. Images were taken at x 63 magnification.

A

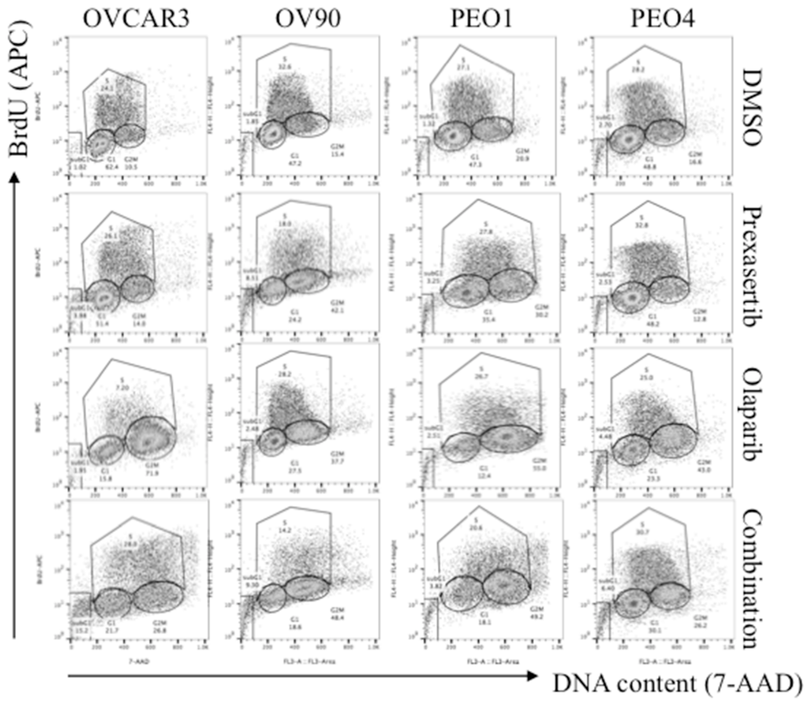

B

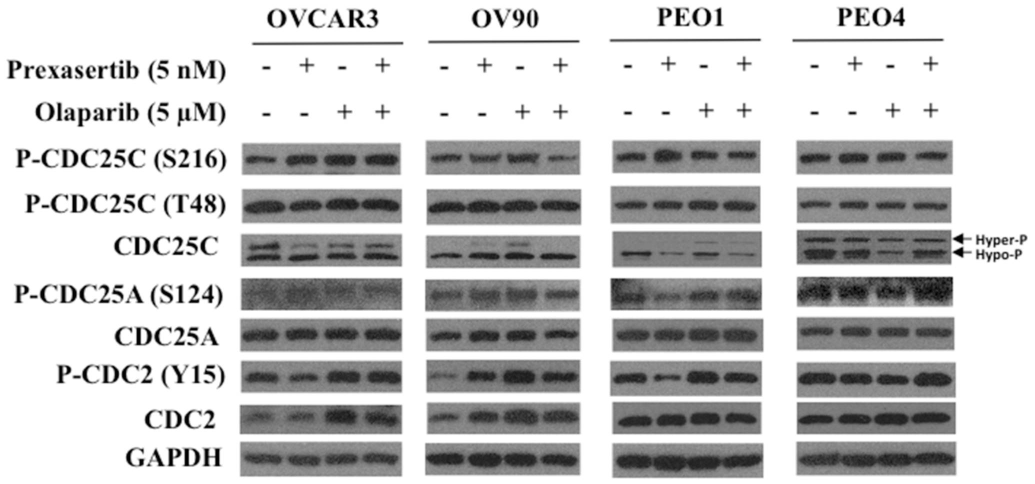

Supplementary Figure 3: Effects of prexasertib and olaparib on the cell cycle and cell cycle checkpoint mediating proteins. (A) Representative profiles of cell cycle analysis by flow cytometry were shown as dot plots. **(B)** Cells were treated with 5 nM prexasertib, 5 µM olaparib, both or 0.01% DMSO for 48 hours and cell lysates were subjected to immunoblotting to examine cell cycle checkpoint mediating proteins.

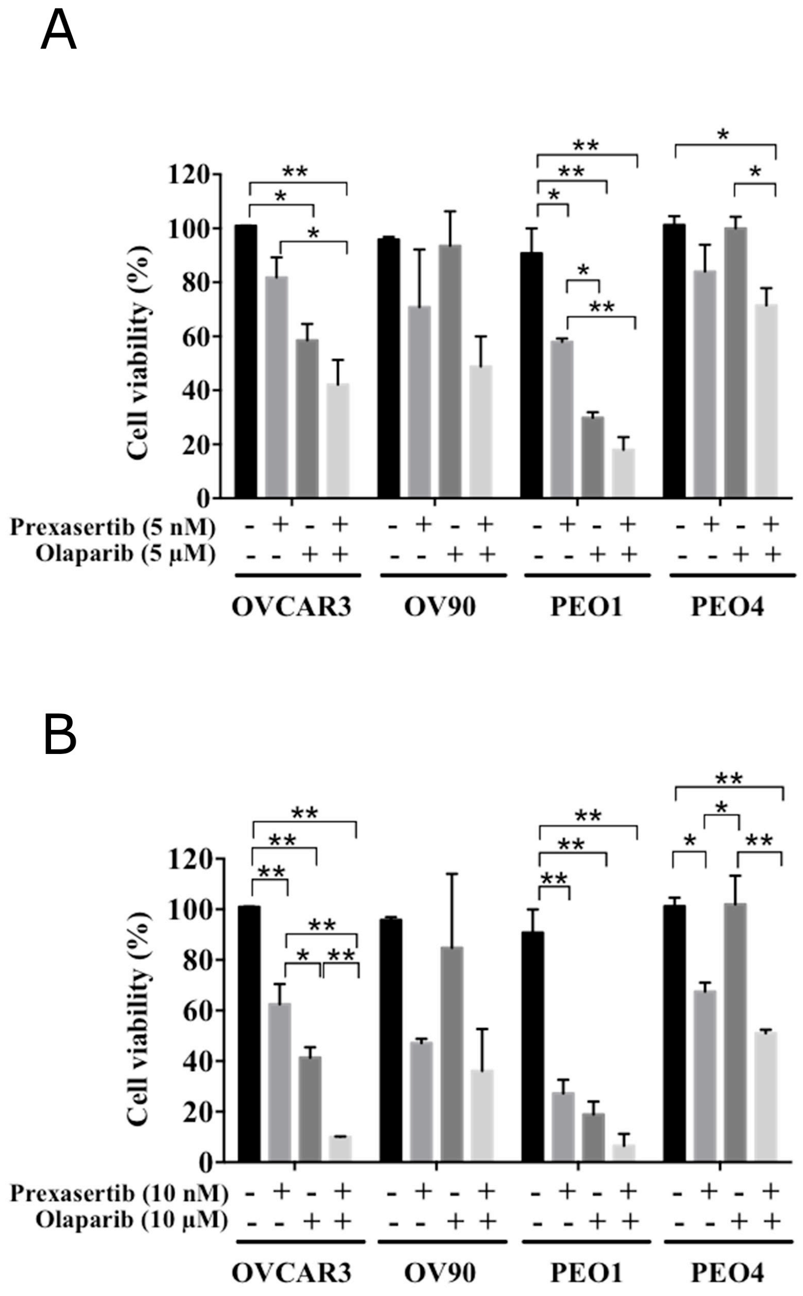

Supplementary Figure 4: Cell viability decreases with increasing doses of olaparib and prexasertib. (A) Cells were treated with either 5 nM prexasertib, 5 µM olaparib, or both for 3 days. **(B)** Cells were treated with either 10 nM prexasertib, 10 µM olaparib, or both for the same duration of time. For both (A) and (B), the cell viability after 3 days treatment was measured by XTT assay, and the data was presented as the mean ± SD from 2 independent experiments. The statistical significance was analyzed using one-way ANOVA. (* = $p < 0.05$, ** = $p < 0.01$).

Supplementary Table 1: CI values for combination concentrations of prexasertib/olaparib. Original Compusyn data for OVCAR3, OV90, PEO1, and PEO4. All cell lines were treated with either olaparib, prexasertib, or both for 3 days. Compusyn was used to calculate combination indices between two drugs, and the complete data set is presented in this table.

See Supplementary File 1
