# Supplementary material for: Prexasertib, a cell cycle checkpoint kinases 1 and 2 inhibitor, increases in vitro toxicity of PARP inhibition by preventing Rad51 foci formation in BRCA wild type high-grade serous ovarian cancer
Source: Oncotarget. 2017 Oct 31;8(67):111026–40. doi: 10.18632/oncotarget.22195 (PMC5762302; doi:10.18632/oncotarget.22195)
Supplement: Supplementary file 2 [file oncotarget-08-111026-s002.docx]

**Supplementary Table 1. CI values for combination concentrations of prexasertib/olaparib**

Original Compusyn data for OVCAR3, OV90, PEO1, and PEO4. All cell lines were treated with either olaparib, prexasertib, or both for 3 days. Compusyn was used to calculate combination indices between two drugs, and the complete data set is presented in this table.

| **OVCAR3** |  |  |  |  | **OV90** |  |  |  |
| --- | --- | --- | --- | --- | --- | --- | --- | --- |
|  |  |  |  |  |  |  |  |  |
| **Olaparib (µM)** | **Prexasertib (nM)** | **Cell Viability** | **CI** |  | **Olaparib (µM)** | **Prexasertib (nM)** | **Cell Viability** | **CI** |
| 1.25 | 1.25 | 0.69 | 0.49 |  | 1.25 | 1.25 | 0.4 | 1.88 |
| 1.25 | 2.5 | 0.71 | 0.72 |  | 1.25 | 2.5 | 0.52 | 1.12 |
| 1.25 | 5.0 | 0.61 | 0.81 |  | 1.25 | 5.0 | 0.29 | 1.61 |
| 1.25 | 10.0 | 0.47 | 0.88 |  | 1.25 | 10.0 | 0.47 | 0.52 |
| 1.25 | 20.0 | 0.15 | 0.49 |  | 1.25 | 20.0 | 0.47 | 1.06 |
| 2.5 | 1.25 | 0.76 | 1.11 |  | 2.5 | 1.25 | 0.43 | 1.98 |
| 2.5 | 2.5 | 0.61 | 0.72 |  | 2.5 | 2.5 | 0.68 | 0.36 |
| 2.5 | 5.0 | 0.61 | 0.99 |  | 2.5 | 5.0 | 0.36 | 0.46 |
| 2.5 | 10.0 | 0.31 | 0.59 |  | 2.5 | 10.0 | 0.42 | 1 |
| 2.5 | 20.0 | 0.11 | 0.4 |  | 2.5 | 20.0 | 0.39 | 1.93 |
| 5.0 | 1.25 | 0.63 | 1.15 |  | 5.0 | 1.25 | 0.99 | 3.15 |
| 5.0 | 2.5 | 0.54 | 0.87 |  | 5.0 | 2.5 | 0.57 | 0.32 |
| 5.0 | 5.0 | 0.42 | 0.75 |  | 5.0 | 5.0 | 0.41 | 0.51 |
| 5.0 | 10.0 | 0.18 | 0.42 |  | 5.0 | 10.0 | 0.48 | 1.12 |
| 5.0 | 20.0 | 0.06 | 0.26 |  | 5.0 | 20.0 | 0.37 | 1.94 |
| 10.0 | 1.25 | 0.42 | 0.93 |  | 10.0 | 1.25 | 0.93 | 0.37 |
| 10.0 | 2.5 | 0.35 | 0.78 |  | 10.0 | 2.5 | 0.74 | 0.44 |
| 10.0 | 5.0 | 0.24 | 0.59 |  | 10.0 | 5.0 | 0.33 | 0.49 |
| 10.0 | 10.0 | 0.1 | 0.35 |  | 10.0 | 10.0 | 0.39 | 1.06 |
| 10.0 | 20.0 | 0.03 | 0.19 |  | 10.0 | 20.0 | 0.6 | 0.2 |
| 20.0 | 1.25 | 0.33 | 1.25 |  | 20.0 | 1.25 | 0.35 | 0.28 |
| 20.0 | 2.5 | 0.29 | 1.14 |  | 20.0 | 2.5 | 0.3 | 0.52 |
| 20.0 | 5.0 | 0.19 | 0.82 |  | 20.0 | 5.0 | 0.57 | 0.24 |
| 20.0 | 10.0 | 0.08 | 0.43 |  | 20.0 | 10.0 | 0.37 | 0.35 |
| 20.0 | 20.0 | 0.02 | 0.21 |  | 20.0 | 20.0 | 0.41 | 0.26 |
|  |  |  |  |  |  |  |  |  |
| **PEO1** |  |  |  |  | **PEO4** |  |  |  |
|  |  |  |  |  |  |  |  |  |
| **Olaparib (µM)** | **Prexasertib (nM)** | **Cell Viability** | **CI** |  | **Olaparib (µM)** | **Prexasertib (nM)** | **Cell Viability** | **CI** |
| 1.25 | 1.25 | 0.48 | 0.93 |  | 1.25 | 1.25 | 0.93 | 0.62 |
| 1.25 | 2.5 | 0.49 | 1.18 |  | 1.25 | 2.5 | 0.99 | 7.12 |
| 1.25 | 5.0 | 0.31 | 0.94 |  | 1.25 | 5.0 | 0.94 | 0.93 |
| 1.25 | 10.0 | 0.13 | 0.77 |  | 1.25 | 10.0 | 0.88 | 12.99 |
| 1.25 | 20.0 | 0.04 | 0.6 |  | 1.25 | 20.0 | 0.82 | 0.33 |
| 2.5 | 1.25 | 0.43 | 1.36 |  | 2.5 | 1.25 | 0.95 | 1.77 |
| 2.5 | 2.5 | 0.43 | 1.56 |  | 2.5 | 2.5 | 0.89 | 0.71 |
| 2.5 | 5.0 | 0.22 | 0.89 |  | 2.5 | 5.0 | 0.96 | 2.65 |
| 2.5 | 10.0 | 0.11 | 0.76 |  | 2.5 | 10.0 | 0.82 | 0.43 |
| 2.5 | 20.0 | 0.05 | 0.75 |  | 2.5 | 20.0 | 0.74 | 0.28 |
| 5.0 | 1.25 | 0.3 | 1.47 |  | 5.0 | 1.25 | 0.76 | 1.1 |
| 5.0 | 2.5 | 0.25 | 1.27 |  | 5.0 | 2.5 | 0.89 | 1.38 |
| 5.0 | 5.0 | 0.15 | 0.86 |  | 5.0 | 5.0 | 0.83 | 0.81 |
| 5.0 | 10.0 | 0.08 | 0.72 |  | 5.0 | 10.0 | 0.76 | 0.52 |
| 5.0 | 20.0 | 0.03 | 0.59 |  | 5.0 | 20.0 | 0.68 | 0.36 |
| 10.0 | 1.25 | 0.18 | 1.43 |  | 10.0 | 1.25 | 0.8 | 1.24 |
| 10.0 | 2.5 | 0.15 | 1.26 |  | 10.0 | 2.5 | 0.71 | 0.73 |
| 10.0 | 5.0 | 0.08 | 0.79 |  | 10.0 | 5.0 | 0.69 | 0.66 |
| 10.0 | 10.0 | 0.03 | 0.46 |  | 10.0 | 10.0 | 0.61 | 0.45 |
| 10.0 | 20.0 | 0.02 | 0.47 |  | 10.0 | 20.0 | 0.54 | 0.34 |
| 20.0 | 1.25 | 0.05 | 0.69 |  | 20.0 | 1.25 | 0.64 | 1.01 |
| 20.0 | 2.5 | 0.05 | 0.65 |  | 20.0 | 2.5 | 0.55 | 0.67 |
| 20.0 | 5.0 | 0.02 | 0.33 |  | 20.0 | 5.0 | 0.55 | 0.68 |
| 20.0 | 10.0 | 0 | 0.01 |  | 20.0 | 10.0 | 0.5 | 0.55 |
| 20.0 | 20.0 | 0 | 0.02 |  | 20.0 | 20.0 | 0.47 | 0.53 |
